# A Mathematical Model of Quorum Sensing Induced Biofilm Detachment

**DOI:** 10.1371/journal.pone.0132385

**Published:** 2015-07-21

**Authors:** Blessing O. Emerenini, Burkhard A. Hense, Christina Kuttler, Hermann J. Eberl

**Affiliations:** 1 Dept. Mathematics and Statistics, University of Guelph, Guelph, ON, Canada; 2 Institute of Computational Biology, Helmholtz Zentrum München, Neuherberg, Germany; 3 Zentrum Mathematik, Technische Universität München, Neuherberg, Germany; Université d’Auvergne Clermont 1, FRANCE

## Abstract

**Background:**

Cell dispersal (or detachment) is part of the developmental cycle of microbial biofilms. It can be externally or internally induced, and manifests itself in discrete sloughing events, whereby many cells disperse in an instance, or in continuous slower dispersal of single cells. One suggested trigger of cell dispersal is quorum sensing, a cell-cell communication mechanism used to coordinate gene expression and behavior in groups based on population densities.

**Method:**

To better understand the interplay of colony growth and cell dispersal, we develop a dynamic, spatially extended mathematical model that includes biofilm growth, production of quorum sensing molecules, cell dispersal triggered by quorum sensing molecules, and re-attachment of cells. This is a highly nonlinear system of diffusion-reaction equations that we study in computer simulations.

**Results:**

Our results show that quorum sensing induced cell dispersal can be an efficient mechanism for bacteria to control the size of a biofilm colony, and at the same time enhance its downstream colonization potential. In fact we find that over the lifetime of a biofilm colony the majority of cells produced are lost into the aqueous phase, supporting the notion of biofilms as cell nurseries. We find that a single quorum sensing based mechanism can explain both, discrete dispersal events and continuous shedding of cells from a colony. Moreover, quorum sensing induced cell dispersal affects the structure and architecture of the biofilm, for example it might lead to the formation of hollow inner regions in a biofilm colony.

## Introduction

Bacterial biofilms are microbial communities of same or different species that attach to surfaces, embedded in a self-produced extracellular polymeric matrix, which gives some protection to the sessile cells against hostile environmental factors such as antibiotics or mechanical washout. The adsorption and absorption properties and enhanced mechanical stability of biofilms make them advantageous to environmental engineers, e.g. in wastewater treatment, soil remediation and groundwater protection [1]. On the other hand, biofilms can be harmful, especially if they form on medical implants or natural surfaces in the human body, hence causing serious infections [2–4]. Biofilms can also lead to biocorrosion of drinking water pipes or industrial facilities [5] and contamination in food processing plants, causing food spoilage [6, 7].

Biofilm development can be divided into three distinct stages: (a) reversible initial attachment of cells to the surface (b) growth of the cells into a sessile biofilm colony and (c) dispersal, or detachment, of cells from the biofilm colony into the surrounding aqueous phase, which contributes to biological dispersal and biofilm rejuvenation.

Biofilm dispersal, such as erosion and sloughing, can be passive or active whereas seeding dispersal is always an active process [8]. The latter, also known as central hollowing refers to the release of large number of single cells from inside the biofilm colony [9–11]. Seeding dispersal can be internally triggered, e.g. by enzyme-mediated breakdown of the biofilm matrix [12], production of surfactants which loosen cells from the biofilm [13], or externally triggered, e.g. changes in nutrient availability [14], production of free-radical species [15] and control by quorum sensing systems [16–18].

While many experimental studies of biofilm detachment and dispersal have been conducted and reported in the literature, this remains a challenging topic due to difficulties in biofilm detachment characterization [19, 20]. Mathematical modeling and simulation studies can provide a complementary view, as they allow to distinguish between different detachment mechanisms.

Many bacteria have the ability to produce signaling molecules which play a part in inducing cell-cell communication [21]. This is normally referred to as quorum sensing. It is a system of stimulus and response, usually assumed to be correlated to local population density, but also affected by diffusive and convective transport of chemical signals [22, 23]. Bacterial cells produce and release small amounts of chemical signaling molecules referred to as autoinducers, e.g. *N-Acyl Homoserine Lactones* (AHL) [24] found in gram-negative bacteria. Once a threshold environmental autoinducer concentration level is reached, the bacteria undergo alterations in gene expression which synchronizes collective behavior. This up-regulation of cells typically also invokes signal production at an increased rate.

The role of quorum sensing in dispersal events has been discovered and documented in some experimental studies, e.g. [16, 17, 25, 26]. Quorum sensing controlled dispersion is still a relatively new field in the study of biofilms but of general relevance as it occurs in a number of relevant species as shown in [17] with the assertion that this feature could be important for development of treatment strategies.

Many experimental or modeling studies focus on biofilm growth, dispersal or quorum sensing induction. We are not aware of studies that focus on the interplay of these three aspects of biofilms systems and how they affect biofilm structure, function and dynamics. In an experimental setting, it is difficult and sometimes challenging to separate the effects of different potential causes, e.g. to distinguish between shear induced and quorum sensing induced dispersal. In a mathematical modeling setup, it is easier to isolate particular aspects of a system. For these reasons a modeling study can be a good first step that guides future work, both theoretically and experimentally. In this paper we will formulate a mathematical model for quorum sensing induced biofilm dispersal. We will carry out computer experiments to investigate potential effects of this phenomenon in biofilm growth and structure.

Mathematical models for bacterial biofilms over the years have greatly contributed to our understanding of biofilm processes so far. The first generation of biofilm models were continuum models with a focus on population and resource dynamics. These models are formulated under the assumption that a biofilm can be described as a one-dimensional homogeneous layer, cf [27, 28]. Newer models take the spatially heterogeneous structure of biofilms into account, and are formulated as spatially multi-dimensional models. A large number of mathematical modeling techniques have been proposed to model biofilms, consisting of stochastic individual based models e.g.[29–31], stochastic cellular automata models e.g.[32–34] and a variety of deterministic partial differential equation models e.g.[35–37]. These models differ in the approach used to describe biomass movement and structure, but they all are coupled with diffusion-reaction models for growth controlling substrates. Models such as these are usually complex, mathematically difficult to analyze and often only amendable to computational simulations.

A variety of biofilm models have included detachment processes in some form, often highly simplified. In traditional one-dimensional models, the biofilm detachment rate is typically a function of the biofilm thickness [28, 38]; similarly in some 2D models the detachment rate is correlated with the biofilm geometry [39]. In other models detachment is correlated with the shear stress induced by the flowing bulk liquid in the biofilm system; e.g. [40–43]. Apart from mechanical washout of biofilm, a model that describes biofilm detachment induced by chemical changes and food limitations is the stochastic cellular automation in [14, 44, 45].

Many modeling studies have investigated quorum sensing in planktonic populations and in biofilm systems, e.g [36, 46–50]. Most quorum sensing models in biofilms focus on up-regulation, only few include the effect of quorum sensing on the biofilm dynamics, structure, and function e.g.[22, 51]. Modeling studies have shown that quorum sensing can also induce inter-colony or non-local communication in biofilms [49, 52] and that gene regulation in a particular colony can be affected by the surrounding colonies via signal transport in the aqueous phase. In [53] it was suggested that both effects, local population size assessment (quorum sensing in the strict sense), and long range effects due to signal transport (e.g. diffusion sensing), can be unified in the concept of *efficiency sensing*.

An important first question is, which of the existing biofilm modeling frameworks to choose for our simulation study. One criterion to base our choice is the treatment of biomass. In many models the cell density is assumed to be always at maximum, e.g. [27, 54]. Biofilm expansion results from production of new cells, and cell loss result to shrinkage of biofilm. Alternatively, some models treat the biomass density as a dependent variable, e.g. [33, 37], which allows them to describe biofilm colonies with strong biomass density gradient. More importantly for our purpose, such models will be able to describe hollowing biofilm structure by reduced biofilm density in the interior of such colonies.

Another criterion to distinguish between biofilm models is whether they are stochastic or deterministic. While often a single simulation of a stochastic model seems to be faster than that of a deterministic model, many such simulations are required to obtain reliable averages, which offset the computational speed advantage. Deterministic models, on the other hand have averaging properties built in.

Based on these considerations, the model that we will use as the basis for our study is the single species density dependent diffusion-reaction biofilm that was originally introduced in [37]. It is a deterministic continuum model that treats biomass density as a dependent variable. It has been derived both via a spatially discrete master equation starting from the view point of a biofilm as a spatially structured population [55] and from equations for conservation of mass and momentum, starting from the view point of a biofilm as an incompressible fluid [52].

Since we are interested in the interplay of various colonies in a biofilm community, a two-dimensional representation of the biofilm instead of assuming the biofilm as a homogeneous layer seems appropriate. We will neglect the flow field in the aqueous phase and the shear induced detachment that it causes and focus on quorum sensing induced dispersal alone. Since flow field calculations are in many instances the most time consuming step in biofilm simulations, this will simplify the modeling greatly. We will consider a hydrostatic environment in which nutrients are transported to the biofilm from the aqueous phase by a diffusion gradient.

## Method

### Basic model assumptions

We develop a mathematical model that describes the dynamics of quorum sensing induced bacterial cell dispersal in growing biofilms. The local amount of sessile bacterial cells is expressed in terms of the local volume fraction they occupy in the biofilm [37]. The bacteria that engage in quorum sensing are assumed to switch from a down- to an up-regulated state when the local concentration of the quorum sensing molecule becomes large enough and *vice versa*. We do not explicitly distinguish between down- and up-regulated cells but implicitly: we assume that the autoinducer production rate is controlled by the local autoinducer concentration.

Dispersal of sessile cells is triggered as the local autoinducer concentration increases. The motility of dispersed cells and its dispersal from the biofilm into the aqueous phase is assumed to be governed essentially by Fickian diffusion, following [56], who studied the movement of motile bacteria in biofilms. In the biofilm the diffusion coefficient of the dispersed bacteria is reduced due to the diffusive resistance of biofilm cells extracellular polymeric substances (EPS) which we subsume implicitly in biomass volume fraction. This is a common assumption in biofilm modeling, e.g. [33, 54, 57].

The quorum sensing signal molecules (autoinducers) are dissolved and are assumed to be transported by Fickian diffusion. They diffuse at a reduced rate in the biofilm compared to the aqueous phase, following [58]. The rate of production of signaling molecules is higher by one order of magnitude for up-regulated than for down-regulated cells.

### Governing equations

Putting these aspects and assumptions together, the model describing the biofilm dispersal is formulated as a system of four partial differential equations. The dependent variables are *M*, *N*, *C* and *A*. *M* denotes the volume fraction occupied by sessile cells and subsumes the EPS. *N* denotes the concentration of the motile bacterial cells which are capable of moving into and in the liquid phase; we refer to these as ‘dispersed cells’. *C* denotes the concentrations of the growth controlling nutrient substrate. *A* represents the concentration of the dissolved quorum sensing molecules. The governing equations read

$$\partial_{t}M=\nabla \left(D_{M}\left(M\right)\nabla M\right)+\frac{\mu C}{k_{1}+C}M-k_{4}M-\frac{\eta_{1}A^{n}}{\tau^{n}+A^{n}}M+\frac{\eta_{2}M}{k_{5}+M}N$$ (1)

$$\partial_{t}N=\nabla \left(d_{N}\left(M\right)\nabla N\right)+\frac{\mu C}{k_{1}+C}N-k_{4}N+\frac{\eta_{1}A^{n}}{\tau^{n}+A^{n}}M-\frac{\eta_{2}M}{k_{5}+M}N$$ (2)

$$\partial_{t}C=\nabla \left(d_{C}\left(M\right)\nabla C\right)-\frac{\mu }{Y}\frac{M_{\infty }C}{k_{1}+C}\left(M+N\right)$$ (3)

$$\partial_{t}A=\nabla \left(d_{A}\left(M\right)\nabla A\right)+\gamma \left(C\right)\;[\alpha +\beta \frac{A^{n}}{\tau^{n}+A^{n}}]\;M_{\infty }\left(M+N\right)$$ (4)

**Fig 1:**
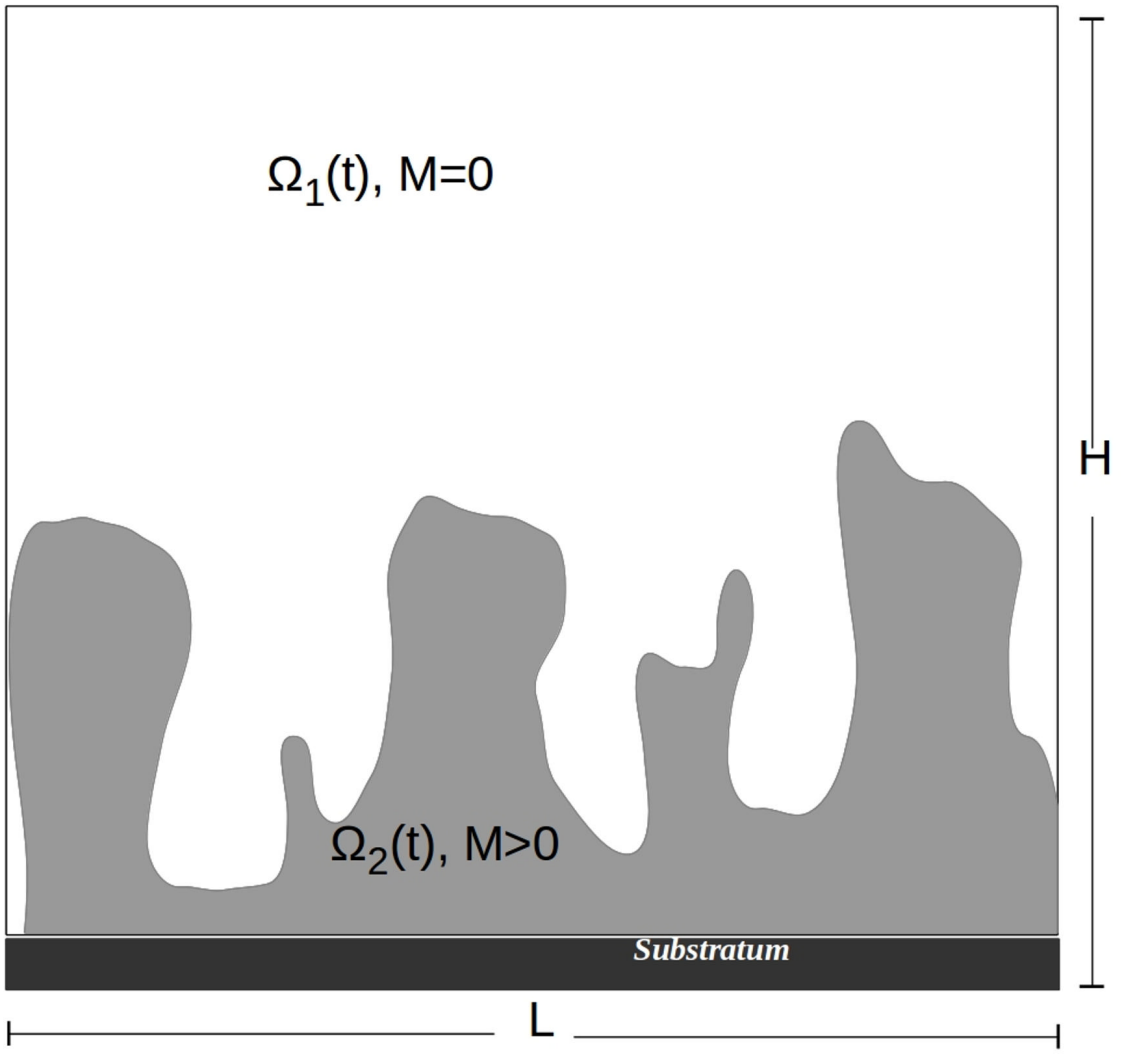
Schematic of the biofilm system. The aqueous phase is the region without biomass present, Ω_1_(*t*) = {(*x*, *y*) ∈ Ω:*M*(*t*;*x*, *y*) = 0}, the biofilm phase is the region with biomass present Ω_2_(*t*) = {(*x*, *y*) ∈ Ω:*M*(*t*;*x*, *y*) > 0}. These regions change over time as the biofilm grows. Biofilm colonies form attached to the substratum, which is a part of the boundary of the domain.

Eqs (1) and (2) describe the growth and spatial movement of sessile and dispersed biomass, *M* and *N*. They are directly coupled by the dispersal and re-attachment terms. The third equation describes the consumption of nutrients by *M* and *N* while the fourth equation describes the production of the quorum sensing molecules. These equations are defined in a domain Ω ⊂ ℝ^*d*^, *d* ∈ {2,3}. The aqueous phase is the region without biomass present, Ω_1_(*t*) = {*x* ∈ Ω:*M*(*t*, *x*) = 0}, and the biofilm phase is the region with biomass present, Ω_2_(*t*) = {*x* ∈ Ω:*M*(*t*, *x*) > 0} (see Fig 1). These regions change over time as the biofilm grows. They are separated by the biofilm/water interface, Γ(*t*): = ∂Ω_2_(*t*) \ ∂Ω. Neither Ω_1_(*t*) nor Ω_2_(*t*) need to be connected domains. In fact Ω_2_(*t*) will in general consist of several colonies that are separated from each other by water. The substratum, on which the biofilm grows is part of the boundary of the domain Ω. It is impermeable to substrate, autoinducer and biomass and it is not reactive.

The density-dependent diffusion coefficient in Eq (1) that describes the biofilm expansion is formulated according to [37] and given by

$$D_{M}\left(M\right)=\delta \frac{M^{a}}{{\left(1-M\right)}^{b}},\;\text{where}\;a,b>1,\delta >0.$$ (5)

The equation degenerates for *M* = 0 where *D*(0) = 0. For 0 ≈ *M* ≪ 1 we have *D*(*M*) ≈ *δM* ^*a*^, i.e. the biofilm diffusion equation behaves like the porous medium equation. In particular this guarantees a finite speed of interface propagation. For *M* = 1 the diffusion coefficient attains a singularity and blows up. For 0 ≪ *M* ≈ 1 the equation behaves like a super-diffusion equation. In particular, the blow up of the diffusion coefficient guarantees that *M* < 1 if a Dirichlet condition is specified somewhere on the boundary of the domain. This means that the super-diffusion effect guarantees that the maximum possible cell density is never exceeded, independent of biomass production terms [59, 60]. Biomass spreading is much slower than the diffusion of the dissolved substrate [28], thus the biomass motility coefficient *δ* [*m* ^2^ *d* ^−1^] is positive but much smaller than the diffusion coefficients of the *N*, *C* and *A* by several orders of magnitude. The diffusion coefficients for *N*, *C* and *A* in Eqs (2)–(4) depend on *M* as well, although in a non-critical way. For dissolved substances like nutrients and quorum sensing molecules, they are lower in the biofilm than in the aqueous phase [58]. We make the same assumption for dispersed cells. We make a linear *ansatz* that interpolates between the experimentally measurable values of diffusion in water (*M* = 0) and in a fully developed biofilm (*M* = 1), i.e.

$$\left\{ \begin{array}{ccc} d_{N}\left(M\right) & = & d_{N}\left(0\right)+M\left(d_{N}\left(1\right)-d_{N}\left(0\right)\right)\\ d_{C}\left(M\right) & = & d_{C}\left(0\right)+M\left(d_{C}\left(1\right)-d_{C}\left(0\right)\right)\\ d_{A}\left(M\right) & = & d_{A}\left(0\right)+M\left(d_{A}\left(1\right)-d_{A}\left(0\right)\right) \end{array}\right.$$ (6)

Note that *d*_*N*, *C*, *A*_(0) > 0, so that the diffusion coefficients are bounded between two finite values. Hence, diffusion is essentially Fickian, and non-degenerate. Diffusion coefficients are measured in *m* ^2^ *d* ^−1^.

**Fig 2:**
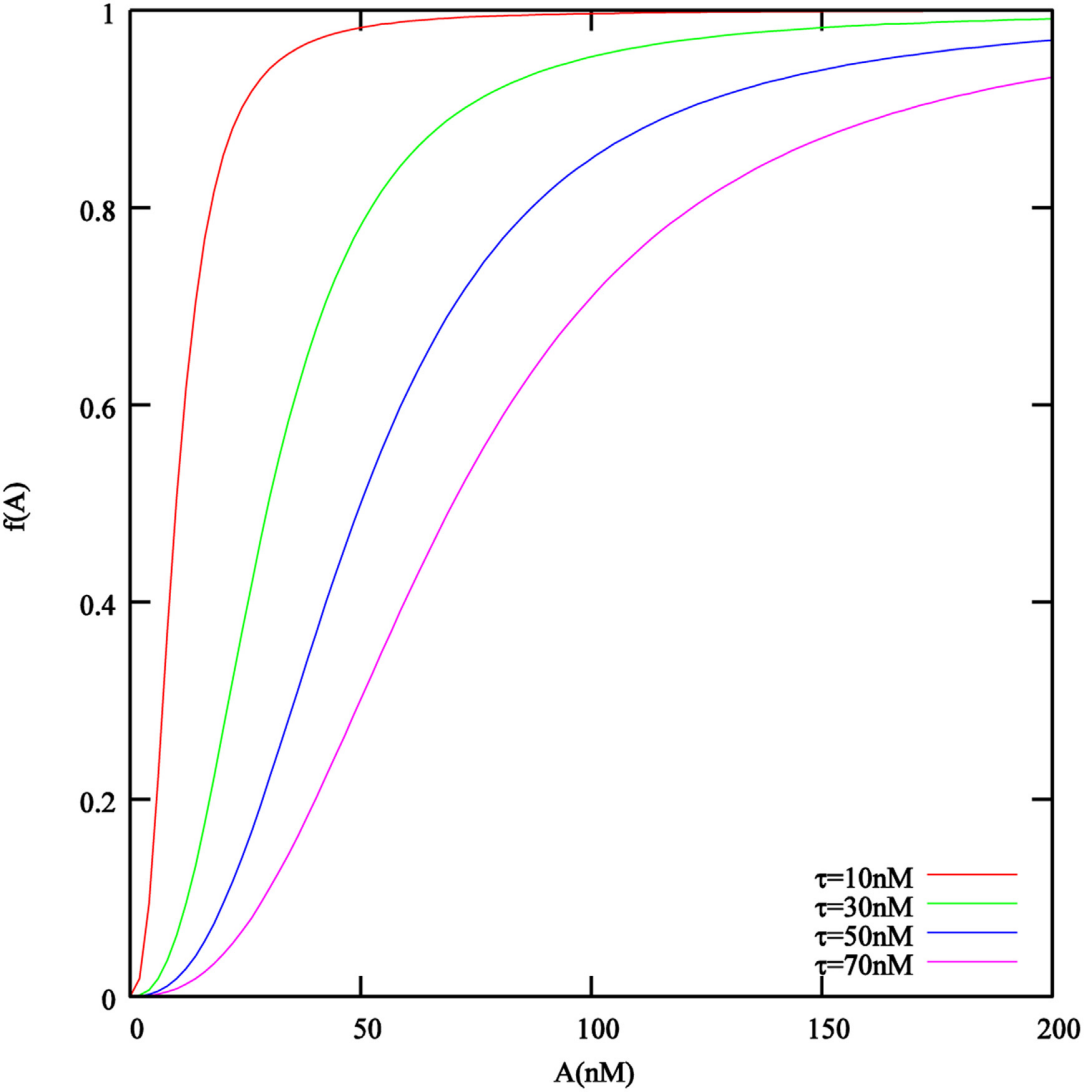
The effect of the local autoinducer concentration *A* on the autoinducer production rate and the dispersal rate is described by the Hill function *f*(*A*) = *A* ^*n*^/(*τ* ^*n*^+*A* ^*n*^). Plotted here for the degree of polymerization *n* = 2.5 that was obtained in [24] and is used in the simulations later on.

The reaction terms and their parameters in Eqs (1)–(4) have the following meaning:

- Growth of sessile and dispersed cells is controlled by the local availability of nutrients in Eqs (1) and (2). This is described by standard Monod kinetics where *k*_1_ [*gm* ^−3^] is the half saturation concentration, and *μ* [*d* ^−1^] is the maximum growth rate. One can argue that the growth rate of the dispersed cells should be different than those of the sessile cells. This would require us to introduce additional model parameters. On the other hand, since dispersed cells diffuse out of the system quickly and, therefore, have only minor effect on the availability of substrate in the system and hence on biofilm growth. Hence, for simplicity, we assume the same growth kinetics for sessile and dispersed cells.
- Cell lysis occurs at the rate of *k*_4_ [*d* ^−1^] in Eqs (1) and (2). For simplicity we assume for both types of biomass the same lysis rate.
- Dispersal of cells from the biofilm is controlled by the local autoinducer concentration. It is negligible if *A* is clearly below the switching threshold *τ* [nM] and attains the maximum dispersal rate *η*_1_ [*d* ^−1^], if *A* ≫ *τ*. The transition between both extreme stages is described by Hill kinetics with exponent *n* [-]. We assume that the dispersal rate is proportional to the autoinducer production rate by up-regulated cells, see below.
- Re-attachment of cells in the biofilm is controlled by the local biofilm density *M*. The re-attachment rate is proportional to *N*for small *M* (relative to *k*_5_ [*gm* ^−3^]) and approximately constant for *M* ≫ *k*_5_, modeled by standard saturation kinetics. In the absence of quantitative data in the literature we assume that the maximum reattachment rate is than the maximum dispersal rate, but smaller. We take it as *η*_2_ = 0.5*η*_1_. This reflects that some of the dispersed cells can re-attach to the biofilm, but that cells that are induced for planktonic life and detached would require a costly up- and down-regulation of many genes to become sessile again.
- Consumption of nutrients in Eq (3) is proportional to the biomass growth rate in Eqs (1) and (2). The proportionality factor is the yield coefficient *Y* [-]. *M*_∞_ [*gm* ^−3^] is the maximum cell density. The compounded parameter *μM*_∞_/*Y* is the maximum consumption rate. Note that the maximum biomass cell density *M*_∞_ does not explicitly occur in the biomass equation because it has been used for scaling when we stated the model in terms of biomass volume fraction instead of densities.
- In Eq (4), autoinducers are produced at a base rate *α* [*nMd* ^−1^ *g* ^−1^ *m* ^3^] if the local autoinducer concentrations is small (relative to induction threshold *τ*) and increases to *α*+*β* where *β* is also measured in [*nMd* ^−1^ *g* ^−1^ *m* ^3^], if it exceeds the induction threshold. The transition is described by a Hill function with exponent *n*. In Fig 2 we plot this function for degree of polymerization *n* = 2.5, as suggested in [24], which we will use in the simulations later on, whereas [61] used a slightly lower value of *n* = 2.2. This function describes a smooth transition between states of no increased autoinducer production (all cells down-regulated) and autoinducer production at maximum rate (all cells up-regulated). It accounts for individual variation between cells. In particular for higher induction threshold values *τ* this implies that a significant amount of autoinducers is already produced at concentrations 0 ≪ *A* < *τ*. A more pronounced switch between both states would be obtained for higher values of *n* than those found experimentally, e.g. [24, 61], see Fig 2. According to experimental studies, the production of quorum sensing signal molecule can be affected by the nutrient, we have included this option in the model as *γ*(*C*) which is described in more detail in S1 Appendix.

We point out that in the absence of quorum sensing activity, e.g. if *α* = *β* = 0 or in the absence of dispersal, e.g. if *η*_1_ = 0, the Eqs (1)–(4) reduces to Eqs (1) and (3), which is the prototype single-species single-substrate biofilm model of [37].

### Computational realization

For our computer simulations we restrict ourselves to the two-dimensional setting with a rectangular computational domain Ω = [0, *L*] × [0, *H*]. The substratum, on which biofilm colonies form is the bottom boundary, *x*_2_ = 0, see also Fig 1. The substratum is assumed to be impermeable to biomass and dissolved substrate, so we pose homogeneous Neumann boundary conditions there i.e. ∂_*n*_ *M* = ∂_*n*_ *N* = ∂_*n*_ *C* = ∂_*n*_ *A* = 0, for *x*_2_ = 0. We consider our rectangular computational domain as part of a larger biofilm reactor. At the lateral boundaries, where *x*_1_ = 0 or *x*_1_ = *L*, we assume a symmetry boundary condition, which allows us to view the domain as a part of a continuously repeating larger domain. Therefore, we pose here as well homogeneous Neumann conditions for all dependent variables i.e. ∂_*n*_ *M* = ∂_*n*_ *N* = ∂_*n*_ *C* = ∂_*n*_ *A* = 0, for *x*_1_ = 0 or *x*_1_ = *L*.

At the top boundary, *x*_2_ = *H*, we pose homogeneous Dirichlet conditions for the biofilm biomass *M*. The degeneracy *D*(0) = 0 in Eq (1) leads to a finite speed of interface propagation in the sense that initial data with compact support imply solutions with compact support. Therefore, as long as biomass does not reach the boundary of the domain, the model satisfies simultaneously homogeneous Dirichlet and Neumann conditions, which are combined in the no-flux conditions *D*(*M*)∇*M* = 0. Since our simulations will be terminated before biomass reaches the top of the domain, the choice of boundary conditions there is not critical. For the nutrient *C*, we pose at the top boundary, *x*_2_ = *H*, an inhomogeneous Dirichlet condition. *C* is set there to the bulk concentration value, which reflects that substrate is added to the system through this segment of the domain boundary. The dispersed cell density and the autoinducer concentration are set there to nil. This enforces a diffusion gradient from the biofilm in the interior of the domain to the boundary and mimics removal of quorum sensing molecules and dispersed cells into the surrounding bulk phase where they are negligible due to instantaneous dilution. Thus we have *C* = *C*_∞_, *M* = *N* = *A* = 0 at *x*_2_ = *H*.

Initially biofilm biomass is placed in small pockets with *M* > 0 at the substratum only. The locations and initial sizes of these pockets will be chosen randomly or explicitly specified *a priori*. Thus ∂Ω_2_(0)∩{*x*_2_ = 0} ≠ ∅, ∂Ω_2_(0)∩∂Ω \ {*x*_2_ = 0} = ∅ and ∫_Ω_2_(0)_ *dx* ≪ ∫_Ω_ *dx*. Ω_2_(0) is typically not connected, i.e. several inoculation sites are usually considered and all have a boundary with *x*_2_ = 0. We will assume that initially no dispersing cells and no autoinducers are in the system, and that the concentration of nutrients is initially at bulk levels, i.e. *C* = *C*_∞_, *N* = *A* = 0 at *t* = 0.

Eqs (1)–(4) are discretized on a regular grid using a cell centered finite difference-based finite volume scheme for space and semi-implicit time-integration, adapted from [57, 64, 65] to account for the new dependent variable *A*, *N*, which are treated in the same manner as *C*. In every time step, four linear algebraic systems are solved, one for each dependent variable. These linear systems are sparse and at least weakly diagonally dominant. They are efficiently solved with the stabilized biconjugate gradient method [66]. The linear solver is prepared for parallel execution on multi-core and shared memory multiprocessor architectures using OpenMP, as described in [65]. Simulations will be terminated when the biofilm reaches a set target size or when a set maximum simulation time is reached. For the visualization of simulation results we use the Kitware Paraview visualization package (spatially resolved plots) and gnuplot (lumped results).

For better interpretation of the computer simulations of the model, the following quantitative lumped measures will be used

- Biofilm size relative to the domain size
  $$\omega \left(t\right):=\frac{\int_{\Omega_{2}\left(t\right)}dx}{\int_{\Omega }dx}$$ (7)
- Average nutrient concentration in Ω_2_:
  $$C_{avg}\left(t\right):=\frac{\int_{\Omega_{2}\left(t\right)}C\left(t,x\right)dx}{\int_{\Omega_{2}\left(t\right)}dx}$$ (8)
- Total sessile biomass in the biofilm:
  $$M_{tot}\left(t\right):=\int_{\Omega }M\left(t,x\right)dx$$ (9)
- The total amount of dispersed cells:
  $$N_{tot}\left(t\right):=\int_{\Omega }N\left(t,x\right)dx$$ (10)
- Average concentration of the quorum sensing molecules in Ω_2_, non-dimensionalized with respect to *τ*:
  $$A_{avg}\left(t\right):=\frac{\int_{\Omega_{2}\left(t\right)}A\left(t,x\right)dx}{\tau \int_{\Omega_{2}\left(t\right)}dx}$$ (11)
- Biomass loss *K*(*T*): This is the relative difference between the net biomass gain and the produced sessile biomass over a period of time *T* defined as follows
  $$K\left(T\right)=\frac{\int_{0}^{T}\int_{\Omega }[\mu \frac{C}{k_{1}+C}]\;Mdxdt-\left[M_{tot}\left(T\right)-M_{0}\right]}{\int_{0}^{T}\int_{\Omega }[\mu \frac{C}{k_{1}+C}]\;Mdxdt}.$$ (12)
  where *M*_*tot*_(*T*) is the amount of biomass in the system at *t* = *T* and *M*_0_ is the amount of biomass initially present in the system.
- The ratio of dispersed cells that are re-attached and the cells that are detached, at time *t*:
  $$Z\left(t\right)=\frac{\eta_{2}}{\eta_{1}}\;[\frac{\int_{\Omega }(\frac{M}{k_{5}+M})\;Ndx}{\int_{\Omega }(\frac{A^{n}}{1+A^{n}})\;Mdx}]$$ (13)
- A measure for the amount of dispersed cells (i.e. the diffusive flux) that left the domain over the time interval [0, *T*]
  $$P\left(T\right)=\int_{0}^{T}\int_{0}^{L}\frac{\partial N}{\partial n}{|}_{y=H}dx_{1}dt$$ (14)

The default model parameters used in the simulations are summarized in Table 1. Parameter values that are varied in the simulations will be stated in the text where the simulation experiments are described.

**Table 1:** Parameter values used in the numerical simulations.

Symbol	Parameter	Source	Value	Unit
*μ*	maximum specific growth rate	[28]	6.0	*d* ^−1^
*Y*	yield coefficient	[28]	0.63	-
*k*_1_	half saturation concentration (growth)	[28]	4.0	*gm* ^−3^
*k*_2_	1st threshold concentration in *γ*_1,2,3,4_	*assumed*	0.05	*gm* ^−3^
*k*_3_	2nd threshold concentration in *γ*_1,2,3,4_	*assumed*	0.1	*gm* ^−3^
*k*_4_	lysis rate	[62]	0.4	*d* ^−1^
*k*_5_	half saturation density (re-attachment)	[28]	0.7	*gm* ^−3^
*M*_∞_	maximum cell density	[62]	10^4^	*gm* ^−3^
*η*_1_	maximum dispersal rate	*assumed*	0.6 − 4.2	*d* ^−1^
*η*_2_	maximum re-attachment rate	*assumed*	0.3 − 2.1	*d* ^−1^
*τ*	quorum sensing induction threshold	[24, 63]	10 − 70	*nM*
*α*	constitutive autoinducer production rate	[24]	0.5520	*d* ^−1^
*β*	induced autoinducer production rate	[24]	5.5200	*d* ^−1^
*n*	degree of polymerization	[24]	2.5	-
*d*_*A*_(0)	diffusion coefficients of *A* (water)	[52]	7.8 × 10^−5^	*m* ^2^ *d* ^−1^
*d*_*A*_(1)	diffusion coefficients of *A* (biofilm)	[52]	3.9 × 10^−5^	*m* ^2^ *d* ^−1^
*d*_*C*_(0)	diffusion coefficients of *C* (water)	[62]	10^−4^	*m* ^2^ *d* ^−1^
*d*_*C*_(1)	diffusion coefficients for *C* (biofilm)	[62]	8 × 10^−5^	*m* ^2^ *d* ^−1^
*d*_*N*_(0)	diffusion coefficients of *N* (water)	*assumed*	10^−4^	*m* ^2^ *d* ^−1^
*d*_*N*_(1)	diffusion coefficients of *N* (biofilm)	*assumed*	2 × 10^−5^	*m* ^2^ *d* ^−1^
*δ*	biomass motility coefficient	[37]	10^−12^	*m* ^2^ *d* ^−1^
*a*	biofilm diffusion exponent	[37]	4.0	-
*b*	biofilm diffusion exponent	[37]	4.0	-
*L*	system length	[62]	4 × 10^−3^	*m*
*H*	system height	*assumed*	1.6 × 10^−3^	*m*
*eps*	asymptote controlling parameter for *γ*_3_ and *γ*_4_	*assumed*	10^−12^	-

## Results

The objective of our numerical simulation experiments will be to better understand quorum sensing induced cell dispersal in biofilm. The primary parameters that we vary in these studies are the threshold parameter *τ* that sets the scale for autoinducer induction, and the maximum dispersal rate *η*_1_ that are most directly linked to this process.

We will first take a look at lumped results, integrated over the computational domain, and then at the local effects on biofilm structure.

### Induction threshold *τ* and erosion rate *η*_1_ control discrete vs continuous dispersal patterns”

**Fig 3:**
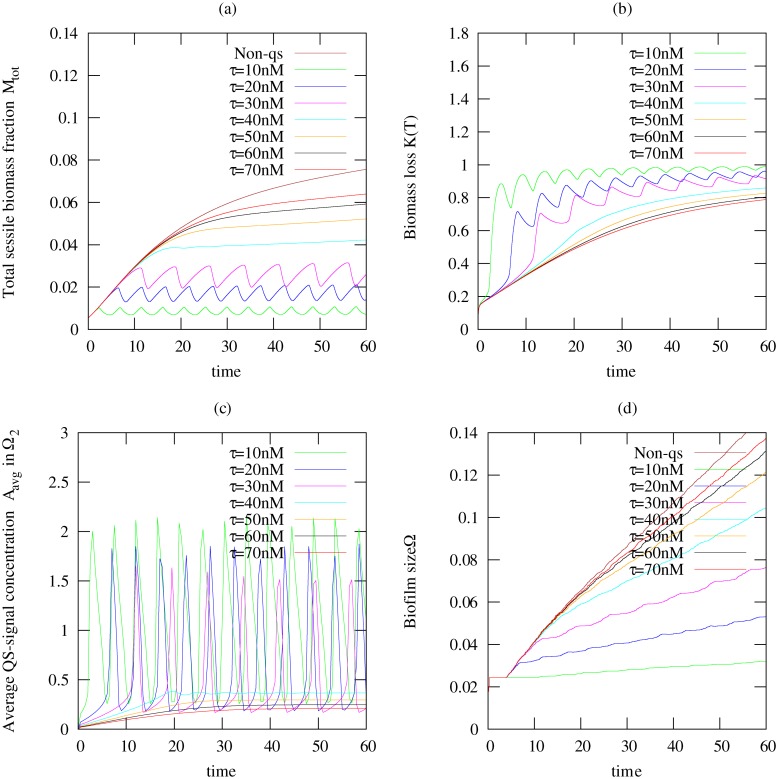
Temporal plots of simulations computed for a non-quorum sensing producing biofilm (Non-QS) and a quorum sensing producing biofilm. Here we used seven different quorum sensing threshold values *τ* = {10, 20, 30, 40, 50, 60, 70}*nM* and fixed maximum dispersal rate *η*_1_ = 3.6/*d*. Shown are (a) the total sessile biomass fraction *M*_*tot*_ in the biofilm, (b) biomass loss *K*(*T*) indicating the amount of biomass that actually dispersed, (c) the average autoinducer concentration *A*_*avg*_ in Ω_2_, and (d) the biofilm size *ω*(*t*).

This first simulation experiments investigates how quorum sensing induced dispersal affects the biofilm growth and dispersal events. Here we have considered a situation where the nutrient concentration has no influence on the quorum sensing signal production, thus *γ*(*C*) ≡ 1. The parameter values listed in Table 1 were used. The quorum sensing threshold parameter was varied within one order of magnitude, *τ* = 10, 20, 30, 40, 50, 60, 70*nM*. The maximum dispersal rate in these simulations was set to be *η*_1_ = 3.6/*d*, smaller than but in the same order of magnitude as the maximum growth rate. The simulations of the quorum sensing induced dispersal model are compared with the results of a non-quorum-sensing-producing biofilm (*α* = *β* = 0). Lumped output parameters of the simulations are plotted in Fig 3. In all cases we see that biofilm growth is sub-exponential, indicating nutrient limitations for growth.

For low values of *τ*, the switching threshold is reached quickly leading to a rapid dispersal of the biomass before the biofilm can grow considerably as shown in Fig 3a for *τ* ≤ 30*nM*. Both dispersed cells and autoinducers are removed quickly from the system. After the first dispersal event, the bacterial cells that are left behind in the biofilm are too few to keep the autoinducer concentration at levels that maintain dispersal, cf Fig 3b and the quorum sensing signal concentration drops, see Fig 3c. The biofilm population starts growing again. A newly increasing amount of biomass in the biofilm means a renewed increase in autoinducers, until these reach threshold and trigger the next dispersal event, and the pattern continues. Overall we see an almost periodic pattern of discrete dispersal events. The biofilm size did not shrink during the dispersal and loss of biomass events as shown in Fig 3d, which implies that the biomass density in the biofilm colonies will reduce.

For higher values of *τ* (*τ* > 50*nM*), the biofilm develops into a stronger colony before the onset of dispersal. Release of cells from the biofilm into the aqueous environment and removal from the system appears continuous and the biofilm population reaches a plateau (see Fig 3a) for *τ* ≥ 50*nM*. The higher the threshold value is, the higher the level at which the biomass plateaus. The results for *τ* = 40*nM* show a transition from the discrete sloughing-like dispersal event to a continuous erosion-like dispersal event.

For all values of *τ*, a substantial amount of the biomass that is produced in the biofilm is dispersed and leaves the system. For low threshold values almost all the produced biomass leaves, while even for high induction points still 90% of the sessile biomass that is produced are lost.

A different growth behavior is observed when the biofilm produces no quorum sensing signal molecule (i.e. *α* = *β* = 0), and thus does not induce dispersal. The biofilm growth for the Non-QS case was limited due to nutrient limitation as seen in Fig 3a and 3d, albeit nutrient limitation is not sufficient enough to induce a leveling off of biomass production but that dispersal balances growth.

We also conducted simulations to investigate the effect of the maximum dispersal rate *η*_1_ on the amount of cells dispersed and on the biofilm, which are reported in detail in S1 Appendix. The results obtained from this investigation reveals that the frequency of the dispersal event changes as *η*_1_ changes. Lower dispersal rates led to a more continuous dispersal event. Increasing the dispersal rate resulted in a more rapid and discrete dispersal event, this is similar to what was observed for small induction threshold.

### A quorum sensing controlled dispersal trigger allows the biofilm to mature before biomass loss

This simulation is carried out to investigate the effect of constant dispersal on biofilm with the aim of answering the question “*If dispersal is important to biofilms, why don’t they shed cells continuously but rely on a mechanism like quorum sensing?*”. For these simulations, the biofilm consists of bacteria that produce quorum sensing signals. By “*constant dispersal*” we refer to the case where the dispersal rate in Eq (1) is kept constant, i.e. does not depend on the autoinducer concentration. We call this also NonQS-induced. In the model, this is the limit case *τ* = 0. This is compared with the situation whereby the dispersal is induced by quorum sensing with *τ* = 50*nM*. The maximum dispersal rate in these simulations is *η*_1_ = 2.4*d* ^−1^. The other parameter values are listed in Table 1.

**Fig 4:**
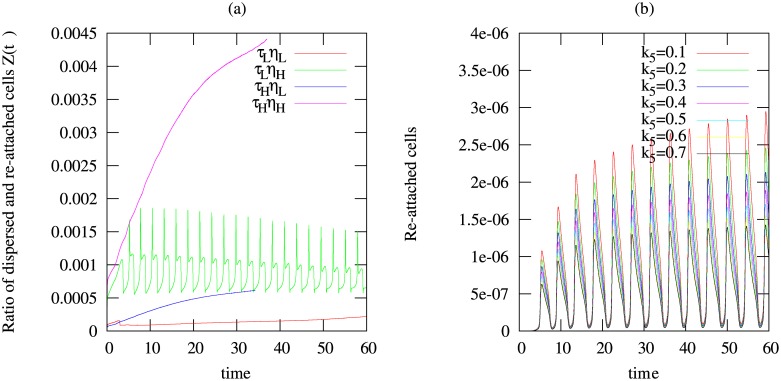
Temporal plots of results for constant dispersal with no influence of quorum sensing using dispersal rates *η*_1_ = 2.4*d* ^−1^, compared to quorum sensing induced dispersal with *τ* = 50*nM*. Shown are (a) the total amount of biomass in the biofilm *M*_*tot*_, (b)the amount of suspended biomass that leaves the biofilm *P*(*T*).

The results from these simulations are presented in Fig 4 for the total sessile biomass, the biofilm size, and the amount of dispersed cells that leave the biofilm. We observe that quorum sensing induced dispersal allows the biofilm to grow before biomass loss is initiated, leading overall to a stronger biofilm, whereas continuous dispersal prevents notable growth of the biofilm.

### Re-attachment of dispersed cells is negligible

Additional simulations were carried out to investigate the influence of the quorum sensing switching parameter *τ* and the maximum dispersal rate *η*_1_ on re-attachment of bacterial cells to the biofilm. In these simulations we have considered four different scenarios consisting of high and low values of *τ* and *η*_1_ respectively resulting from the following combinations: *τ*_*H*_ *η*_1__*H*_, *τ*_*H*_ *η*_1__*L*_, *τ*_*L*_ *η*_1__*H*_ and *τ*_*L*_ *η*_1__*L*_, where index *L* represents ‘Low value’ and index *H* represents ‘High value’. The low and high values of *τ* are *τ* = 10*nM* and *τ* = 70*nM*, respectively, while the low and high values of *η*_1_ are *η*_1_ = 0.6*d* ^−1^ and *η*_1_ = 4.2*d* ^−1^, respectively. We assume here that the production of the signal molecule is not significantly influenced by the nutrient concentration, hence *γ*(*C*) ≡ 1. Every other parameter used for the simulation is as listed in Table 1.

**Fig 5:**
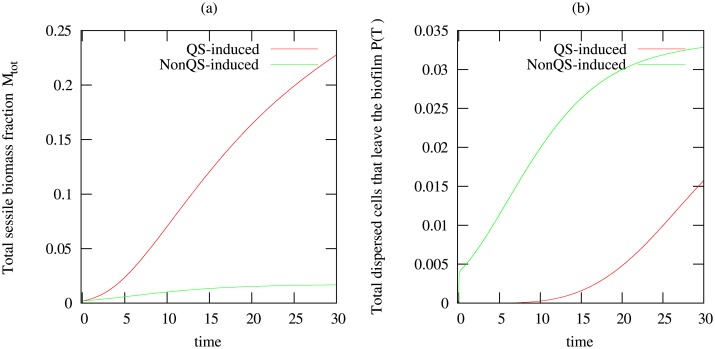
Temporal simulations results to investigate the re-attachment of bacterial cells after dispersal. Shown are (a) the ratio of dispersed cells that are re-attached and the cells that are detached Z(t) using *τ*_*L*_ = 10*nM*, *τ*_*H*_ = 70*nM*, *η*_1__*L*_ = 0.6/*d*, *η*_1__*H*_ = 4.2/*d*; (b) amount of re-attached cells defined by $\eta_{2}\frac{M}{k_{5}+M}$ and computed for different values of *k*_5_ i.e. *k*_5_ = 0.1, 0.2, 0.3, 0.4, 0.5, 0.6, 0.7*gm* ^−3^ using the quorum sensing threshold value *τ* = 10*nM* and the maximum dispersal rate *η*_1_ = 3.6*d* ^−1^.

By computing the ratio of the dispersed and re-attached cells *Z*(*t*) as shown in Fig 5a, we found that re-attachment is generally negligible compared to the amount of cells dispersed irrespective of the choice of *τ* and/or *η*_1_.

Another parameter that controls re-attachment of cells is *k*_5_ seen in Eqs (1) and (2) describing the attraction of bacterial cells towards the biofilm. By setting *τ* = 10*nM* and *η*_1_ = 3.6*d* ^−1^, we compare the amount of re-attached cells defined by $\eta_{2}\frac{M}{k_{5}+M}$ for different values of the parameter *k*_5_ varied over one order of magnitude, *k*_5_ = 0.1, 0.2, 0.3, 0.4, 0.5, 0.6, 0.7*gm* ^−3^. We observe also here that re-attachment is negligible (see Fig 5b).

### Quorum sensing controlled dispersal can explain central hollowing

From the lumped output parameters in Fig 3a and 3c we observe that during a discrete dispersal event and the following biomass growth period the biofilm size remains constant. This suggests that biomass loss due to dispersal does not lead to a shrinking of the colony but to a decrease in local cell density in the biofilm.

**Fig 6:**
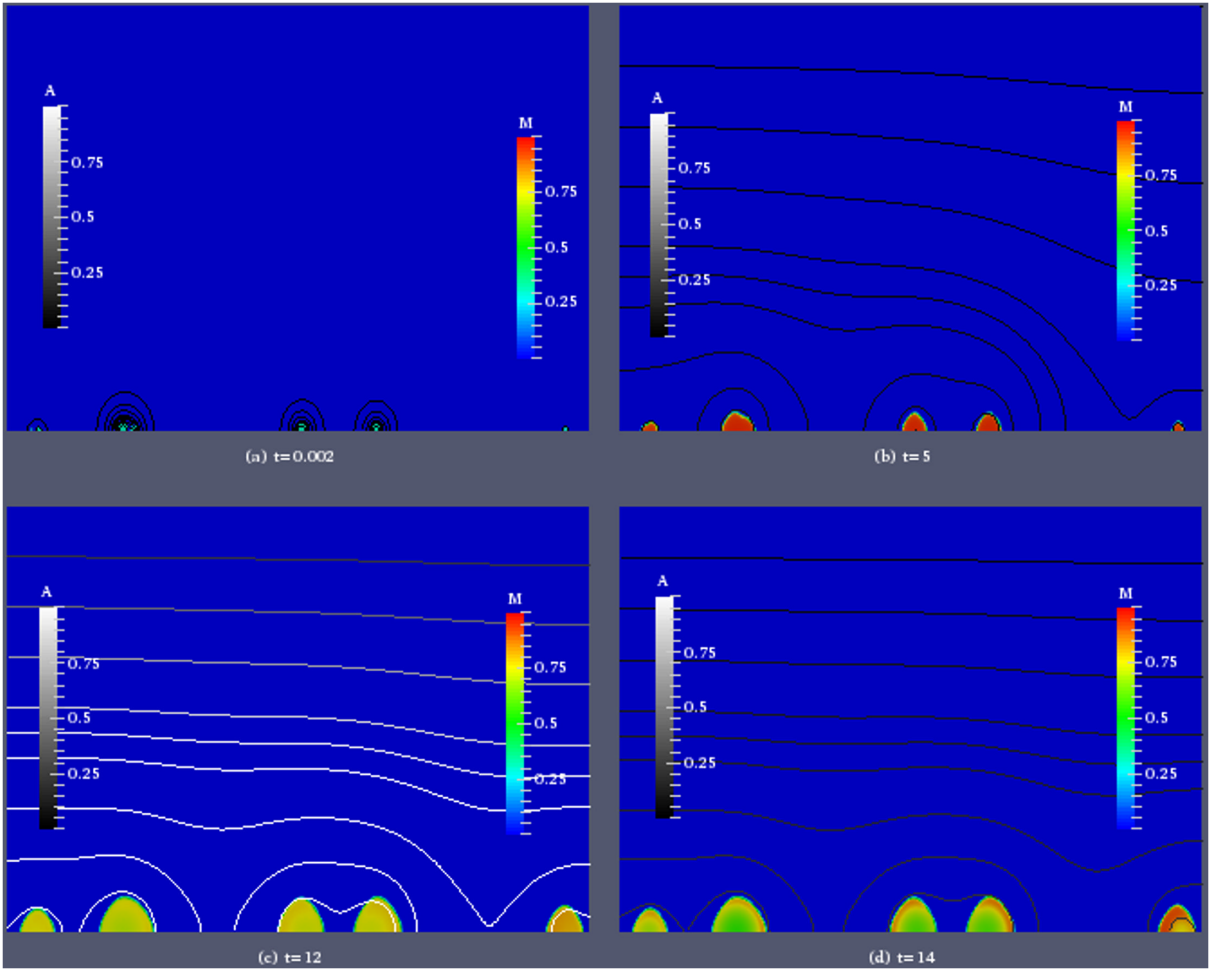
Simulation of biofilm growth for induction threshold *τ* = 20*nM* and maximum dispersal rate *η*_1_ = 3.6*d* ^−1^. Color coded is the biomass density *M*, iso-lines of the autoinducer concentration *A* are plotted in grayscale.

**Fig 7:**
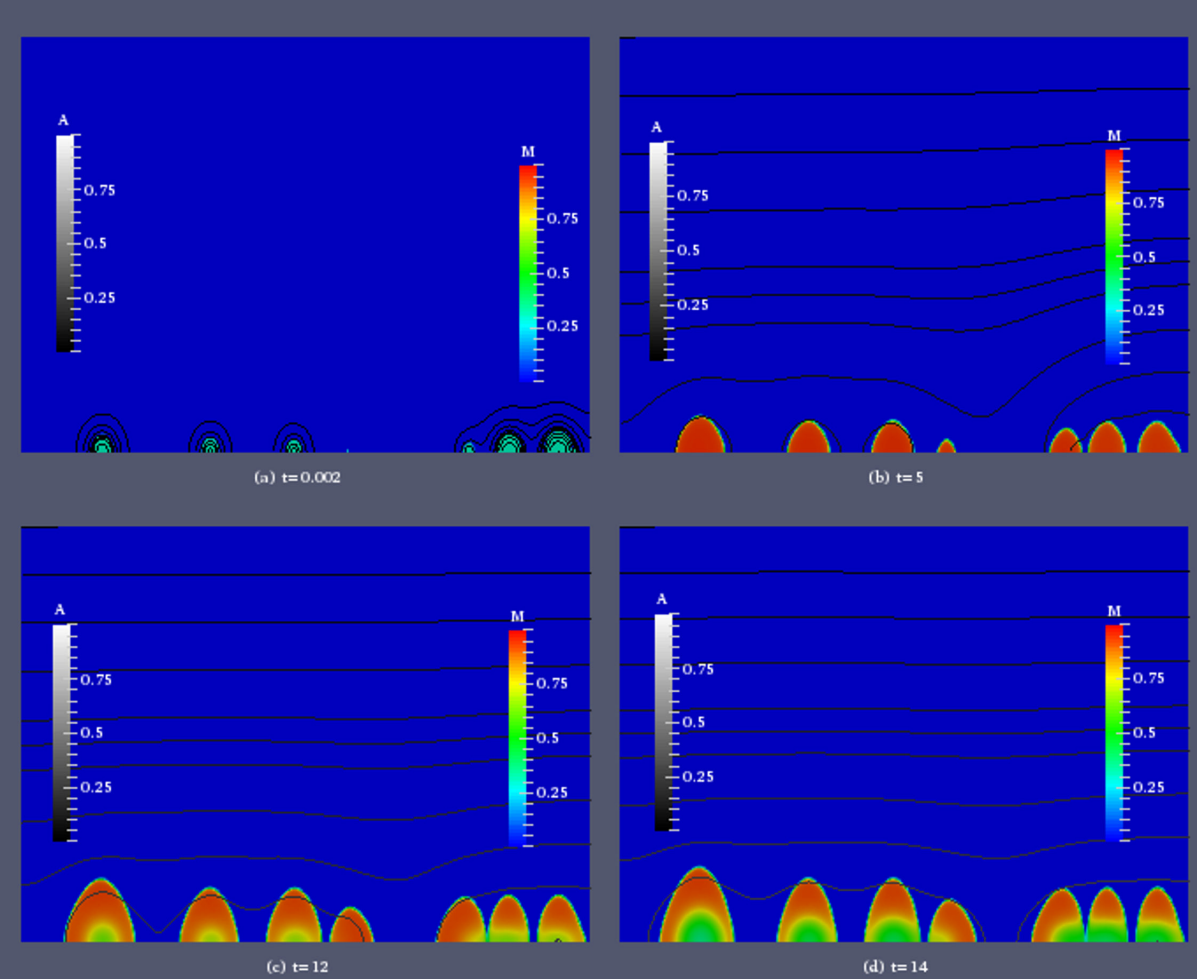
Simulation of biofilm growth for induction threshold *τ* = 60*nM* and maximum dispersal rate *η*_1_ = 3.6*d* ^−1^. Color coded is the biomass density *M*, iso-lines of the autoinducer concentration *A* are plotted in grayscale.

To investigate the effect of quorum sensing triggered dispersal on the spatial structure of the biofilm colonies and the local biomass distribution in more detail, we visualize two simulations, one in which discrete dispersal events are observed, with *τ* = 20*nM*, *η*_1_ = 3.6/*d* (Fig 6), and one in which dispersal appears continuous, with *τ* = 60*nM*, *η*_1_ = 3.6/*d* (Fig 7). We show for selected time instances the spatial distribution of the sessile biomass *M* and iso-lines of the autoinducer concentration. In both cases we use the same initial distribution of biomass. Six colonies are randomly placed on the substratum. They differ in size, but the biomass density inside each colony is initially set to be 0.3.

In the case of the lower threshold value *τ* in Fig 6, immediately after the simulation starts, *A* is very small. At the next shown time instance *t* = 5 the biomass density inside the colonies has reached values close to maximum density and expansion of the biofilm has started. The colonies have grown in size, a small and a large colony that were initially placed close together have merged. Shortly after, the first dispersal event was initiated which leaves the colonies with lower biomass density in the inner cores than in the outer rims. This structural change is more conspicuous in the next snapshot at *t* = 12 which is taken shortly after the second dispersal event. The colonies have still the same size as before, but in their inner core the biomass density is substantially decreased. An exception is the smaller colony in the middle of the domain, in which the biomass density is larger than in the neighboring colonies. This is explained by lower autoinducer concentrations there that have not triggered a discrete dispersal event. Due to the smaller amount of biomass in the system overall and removal of the signal from the system due to diffusion, the autoinducer concentration has dropped again below the threshold value. At the last time instance that we show, *t* = 14, the biomass inside the colonies has increased again, without a notable increase in colony size. In the smallest colony in the center of the domain, in which no dispersal event has taken place the biomass distribution is more homogeneous.

We contrast this with the result for the higher threshold value *τ* = 60*nM* in Fig 7. Immediately after the simulation starts, at *t* = 0.0002, the situation is the same as in Fig 6. At *t* = 5 The colonies have grown with a rather homogeneous distribution of biomass. The small and large colony that were originally in close proximity have merged. The signal concentration is still well below threshold with a maximum value of *A* ≈ 0.082*τ*. Accounting for the different threshold concentrations values, this is a value similar, but lower than at the same time in Fig 6. At *t* = 15, the colonies have increased considerably in size, but in their inner regions the biomass density is much smaller than in the outer layers. The autoinducer concentration has increased as well, due to the larger amount of biomass. Although it is remains clearly below the threshold value, substantial cell dispersal has started. Since the signal concentration are highest inside the colonies, cells are lost there at a higher rate than in the outer layers. This is explained by Fig 2, which shows that for such high threshold values even for signal concentrations clearly below *τ* the dispersal rate can be substantial. This patterns continues, as time progresses. For *t* = 20, we observe an increase in the size of the colonies, with an outer rim with high biomass density. The biomass density in the inner core decreases as the biofilm increases, leading to regions with fewer cells, i.e hollowing structures, as reported in experimental studies, e.g. [44, 67].

We observe hollowing of biofilm colonies both for simulations with discrete and continuous cell dispersal, but there is a substantial difference. In the case of discrete dispersal events, the biomass density increases after the event, filling up the hollows. In the case of continuous dispersal loss, biomass is not replenished inside the colonies, rather the outer layers expand and the colony increases in size quicker, see also Fig 3d. Hollowing occurs because the autoinducer concentration is highest in the inner layers of the colony, due to the maximum principle for diffusive systems, e.g. [68]. This implies that cells there up-regulate and disperse first.

### Nutrient dependence of the autoinducer production rate has only minor effect on dispersal

In this simulation experiment, we investigated the influence of nutrient availability on the production of quorum sensing signal molecule which was included in our model (Eq (4)) as an option controlled by the function *γ*(*C*). The description of *γ*(*C*) and the details of the simulation experiment and the results can be found S1 Appendix. The effect of nutrient concentration on the production of the autoinducer signal molecule was investigated in two scenarios namely: biofilm and microfloc. For the biofilm case, high nutrient concentrations are observed only initially and decline quickly as the the biofilm grows. In the case of a microfloc, there is a decreased autoinducer production during high concentration than when the nutrient concentration is low. In summary we observe that the influence of nutrient availability on the autoinducer production rate, as tested here, does not have much effect on cell dispersal.

## Discussion

### Modification of biofilm growth and dispersal

Our study presents, to our knowledge, the first theoretical model analyzing interaction between quorum sensing and dispersal in biofilms with respect to their effects on biofilm structure and population dynamics. The simulation results indicate that this interplay affects both the structure and thickness of existing biofilm colonies as well as the cell dispersal, i.e. the potential to colonize new habitats. The development of hollows, i.e. areas with very low cell densities within biofilms, and the potential to generate fluctuations of biofilm thickness, autoinducer concentration, cell dispersal and re-attachment are predicted by our model. The phenomenon of fluctuations in biofilms has been known before, but not necessarily discussed in the context of quorum sensing, e.g. [14, 69]. Hollows caused by dispersion or other mechanisms are well-known in biofilms. The hollows within the colonies, which are predicted by our study, resemble the voids reported as a result of agr-QS induced detachment in an experimental study of *Staphylococcus aureus* colonies [70]. After detachment of the induced cells only a shell of non-induced cell remains, until the growing colony enters a new cycle of induction and detachment. On the biofilm scale, this can translate into waves of detachment and re-growth, which is connected with oscillations in biofilm mass and (effective) -thickness. It was reported in [71] that such periodic detachment was mediated by quorum sensing induced surfactant production in *S. aureus* and speculated that this is a widespread mechanism in the bacterial world. Other released factors such as quorum sensing controlled exoprotease also have been found to promote detachment [72]. Generation of voids in biofilms or colonies caused by quorum sensing induced dispersal also occurs in other species, such as *Pseudomonas aeruginosa*[73]; [74] reported a rather continuously dispersal behavior in a *P. aeruginosa* biofilm.

Our study showed how cells principally can modify a number of critical traits such as biofilm thickness, -biomass and fraction of dispersed cells by shifting QS threshold and dispersal rates. Even more, such shifts can switch dynamics of dispersal, biomass growth and autoinducer concentration between more continuously and discrete; the latter resulting in an oscillatory behavior. In case of such periodicity, amplitude and frequency can be changed via the same regulated parameters. In fact, experimental studies showed that cell dispersal rates can be promoted by environmental factors like nutrient depletion [14, 75, 76]. Similarly, environmental stresses like starvation often result in a promotion of quorum sensing induction [53]. This can be achieved by regulation of autoinducer receptor or autoinducer synthase, which directly or indirectly corresponds to a shift of the threshold for induction [77–82].

### Ecological relevance of biofilm and dispersal parameters

Thickness and biomass have significant impact on the ecological functionality of biofilms. Growth in biofilms protects bacteria from environmental challenges such as antibiotica or other toxic substances, immune response in hosts, grazing stress from *protozoa*, and mechanical washout. Furthermore, it facilitates cooperation between cells. Most of these aspects are promoted with increasing biofilm thickness. Contrarily, competition for resources, such as nutrients, and waste accumulation impede growth of populations in biofilms of increasing thickness.

For colonization of new habitats bacteria in biofilms usually enter the planktonic state as single cells or in smaller groups with little EPS protection. The death rate in plankton is higher. Thus the decision between planktonic and biofilm states is a trade-off. It depends on a number of factors such as nutrient supply and pressure by competitors or predators, and therefore needs to be controlled by the cells, in order to promote fitness by keeping the biofilm at an optimal size. Our simulations indicate that biofilm dispersal tied to quorum sensing is a mechanism by which the biofilm could achieve this. Integration of nutrient or other stress aspects into the information carried by autoinducers probably allows the cells in a population for a coordinate response to environmental challenges, optimized with respect to efficiency under the actual habitat conditions [53, 83].

It has been shown experimentally that the vast majority of cells produced in a biofilm will eventually detach and enter the aqueous phase [84, 85] and it was suggested that being a “cell nursery”, i.e. a source of planktonic cells, is one of the functions of biofilms [86]. Our results are in agreement with this as we have seen that even maximum dispersal rates much smaller than maximum growth rates can lead to substantially more than 90% of cells detaching, both in a periodic or in a continuous dispersal mode. Moreover, experiments have indicated that dispersal of cells into the aqueous phase can occur at all stages of biofilm development, including small colonies that still grow or larger fully established colonies [15, 85, 87]; our simulations show that this is compatible with quorum sensing controlled dispersal which, regulated by parameters, can be observed for small and large colonies.

The outcome that the systems can change between continuous and oscillating behaviour by shifting one or a few controlled parameter values has some interesting ecological implications. Larger oscillatory detachment events will periodically move the biofilm thickness away from the optimal values with respect to the above mentioned fitness trade-off. On the other hand, such a coordinated detachment strategy might save costs for each involved cell, e.g. for the production of surfactants required for the removal from the biofilm matrix, and minimize loss by predators as known for mass events in *eukaryotes*. Although it is intriguing to assume that this degree of freedom is used by the cells to optimize behavior in a way dependent on the environmental conditions to optimize the fitness, this has to be confirmed experimentally yet.

While detachment and dispersal are central features for the fitness and thus quite well investigated, relatively little is known about the dynamics and the underlying mechanisms. Although experimental studies with different species reported continuous and oscillatory dispersal behavior, to the best of our knowledge none investigated whether and under which conditions both can occur in the same species. Thus the model presented here gives some valuable indications. Although our model focuses on quorum sensing and dispersal, the outcome has an impact on a variety of other bacterial traits. Usually a bacterial quorum sensing system does not regulate just a single gene or phenotype, but up to several hundred genes and consequently a variety of phenotype aspects, including interaction with potential hosts [88–91]

Thus, the strong effect of the interplay of quorum sensing and dispersal on the quorum sensing dynamics is directly connected with bacterial properties such as virulence in humans, animals or plants, but also to beneficial activities in other bacterial species. In summary, the interplay of quorum sensing and detachment under the influence of nutrients affects growth dynamics, structure and function of biofilms. Regulating this interplay adds a degree of freedom to bacterial biofilms in response to environmental conditions.

### Evolutionary advantage of QS regulated dispersal

Although a thorough evolutionary analysis is beyond the scope of our study, it provides some hints to answer the question why QS control of dispersal might be advantageous. Beside the potential fitness benefits discussed above, i.e. keeping biofilm growth/thickness and dispersal in an optimized balance, this control design enables a young colony or biofilm to focus first on growth, i.e. to maximize the protection rendered by attached growth in EPS matrices as fast as possible Fig 4. Note that such a behavior could, in principle, also be reached by a more direct control of dispersal e.g. in dependency on nutrient depletion. Quorum sensing control allows for an integration of the specific information of each cell at its specific side in a spatially structured communication, enabling a response optimized rather with respect to the situation of each cell within the entire population than to the situation of isolated cells [83]. This is of special relevance in spatially structured populations, as e.g. cells in lower layers of the biofilm may be exposed to stronger nutrient stress, potentially resulting in an up-regulation of autoinducer production. As a result, the cell is enabled for a contextual interpretation of the state of the neighbouring cells relative to its own. Furthermore, QS control of dispersal promotes synchronization of response within the population, as seen e.g. in the oscillatory behavior.

### Treatment consequences

Understanding the mechanisms that control biofilm dispersal and quorum sensing dynamics is of crucial interest from the human perspective, e.g. to develop and optimize treatment strategies which consider or even exploit this interplay. As quorum sensing is a master regulator of virulence in most known pathogens, strategies for an efficient suppression are highly desirable and have been proposed as an alternative for antibiotics [51, 92]. On the one hand, promotion of detachment by other, non-quorum-sensing related inductors would help to diminish or avoid virulence and, by limiting or even decreasing biofilm thickness, could promote effectiveness of antibiotic treatment. Detached, i.e. planktonic cells are assumed to be more vulnerable for antibiotica treatment, e.g. [75]. If a sufficiently high dose of antibiotics is supplied, a treatment which shifts the population towards the planktonic state will probably support an eradication of the infection [86].

To go even a step further, shifting the detachment towards an oscillating behavior might be another or an additional strategy, as thinner biofilms, which periodically emerge, combined with a larger fraction of cells in the planktonic state, could be more susceptible to antibiotics. Possible treatments include drugs which directly affect quorum sensing systems and/or detachment e.g. by blocking or mimicking autoinducers. Such treatment strategies are currently under development (see e.g. [93]). Our study indicates that in future more indirect treatments, e.g. via modulating nutritional or stress conditions, could aim at mechanisms which down-regulate quorum sensing threshold or up-regulate dispersal.

Antibiotic treatments might be associated with additional effects relevant for signaling, which are not regarded in our model, e.g. emergence of layers of dead cells possibly interfering with signal diffusion, and up-regulation of quorum sensing activity by low sublethal drug concentrations which might occur in lower parts of a biofilm [79, 94]. The net effect of such new strategies should be estimated by a combination of experimental and mathematical modeling studies. Thus, a detailed knowledge about the interplay of quorum sensing, dispersal and nutrients is of high interest. Our study attempts to be a first step for such new and promising strategies.

## Conclusion

In summary, our *in silico* experiments suggest the following conclusions:

- Dispersal of cells from the biofilm into the aqueous environment balances growth of the biofilm and is important for downstream colonization. Coupling the dispersal rate to quorum sensing provides the opportunity for the biofilm colony to first invest in itself and to grow to a certain community size before shifting to a mode of producing cells for downstream proliferation.
- A single quorum sensing based mechanism can explain both, periodic dispersal in discrete events and continuous dispersal, depending on parameters. It can also, and again in dependence of parameters, explain cell dispersal from smaller and larger colonies. It also provides a potential mechanism for the biofilm to regulate dispersal in dependence of its size and to ensure a certain colony strength.
- Surface attached microcolonies of biofilms undergo internal changes during seeding dispersal. Quorum sensing induced cell dispersal may affect the structure and architecture of the biofilm and can leave behind “hollow” shell-like structure which are less dense with few cells inside. In this process of dispersal, only very few of the dispersed cells get re-attached to the biofilm, hence we conclude that the re-attachment of dispersed bacterial cells is very negligible.
- Quorum sensing triggered seeding dispersal can lead to a substantial amount of cells produced in a biofilm under protected conditions to be shed into the environment. This supports the notion that biofilm act as cell nurseries in the facilitation of downstream colonization.
- Interfering with the quorum sensing mechanism and enhancing dispersal might make the biofilm more vulnerable to antibiotics; on the other hand suppressing quorum sensing might make the biofilm more susceptible to mechanical removal and slow down the biofilm’s potential for downstream colonization

## Supporting Information

S1 Appendix Additional simulation results. (PDF)
