# Supplementary material for: A Mathematical Model of Quorum Sensing Induced Biofilm Detachment
Source: PLoS One. 2015 Jul 21;10(7):e0132385. doi: 10.1371/journal.pone.0132385 (PMC4511412; doi:10.1371/journal.pone.0132385)
Supplement: S1 Appendix — (PDF) [file pone.0132385.s001.pdf]

S1 Appendix: Additional Simulation Results.

We present here the details of additional simulation results, the implications of which are discussed in the main text. We refer the reader to the main text for model description, definition of output parameters, and simulation parameter values.

Biofilm growth and dispersal

We conducted more simulation experiments to investigate how quorum sensing induced dispersal affects the biofilm growth and dispersal events when the maximum dispersal rate η_1 is varied. We considered a situation where the nutrient concentration has no influence on the quorum sensing signal production, i.e. $\gamma(C) = 1$. The parameters used are listed in Table 1 in the main text, except for the dispersal rate η_1, which was varied as $0.6, 1.2, 1.8, 2.4, 3.0, 3.6, 4.2d^{-1}$ and the dispersal threshold concentration $\tau = 10nM$. For low values of η_1, we observe a continuous biomass loss and that the amount of biomass in the biofilm, as well as the autoinducer concentration levels off, as shown in Figs.1a and 1c. The smaller the maximum erosion rates η_1, the higher the plateau will be. Smaller maximum erosion rates lead to larger biofilms, cf Fig.1d.

The lower the dispersal rate is, the more continuous is cell dispersal, even for small value of τ. For higher values of η_1, the dispersal events are rapid and discrete as we have seen before for small induction thresholds (see main text). Again, after each dispersal event, too few bacterial cells are left behind to produce enough autoinducers to maintain dispersal; the signal concentration falls back below threshold, cf Fig.1c and the biofilm starts growing again. When the amount of biomass becomes strong enough for the concentration of the quorum sensing signal molecule to reach threshold, the next dispersal event is triggered, etc. The frequency of the dispersal events changes as η_1 changes, but the amplitude appears to be insensitive to this parameter. In all cases the vast majority of biomass produced in the biofilm is lost, cf Fig.1b.

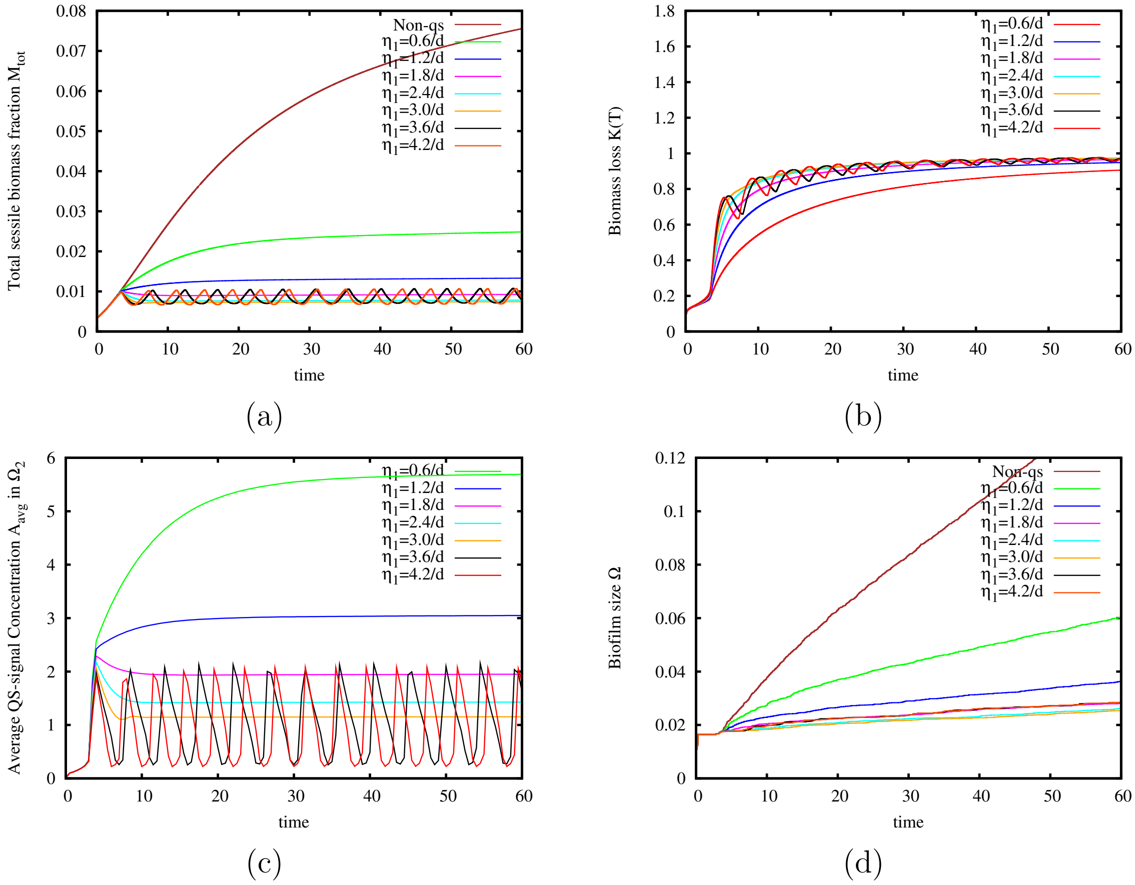

Fig. 1: **Temporal plots of simulations of a non-quorum sensing biofilm (Non-QS) and a quorum sensing biofilm.** Here we used seven different dispersal rates $\eta_1 = \{0.6, 1.2, 1.8, 2.4, 3.0, 3.6, 4.2d^{-1}\}$ and fixed quorum sensing threshold $\tau = 10nM$. Shown are (a) the total amount of sessile biomass in the biofilm, $M_{tot}(t)$, (b) biomass loss $K(T)$ indicating the amount of biomass that actually dispersed, (c) the average autoinducer concentration $A_{avg}(t)$ in Ω_2, (d) the biofilm size $\Omega(t)$.

Nutrient induced autoinducer production

The dependence of the quorum sensing signal production is controlled by the function $\gamma(C)$ which is defined in such a way that $0 < \gamma(C) \leq 1$ where the nutrient concentration C lies between zero and unity. The $\gamma(C)$ is so defined to help capture the effect of nutrient availability on the production of quorum sensing when the nutrient is limited and when it is in abundance respectively. We test for different choices of $\gamma(C)$, depicted in Fig.2. $\gamma_1(C) \equiv 1$ reflects the scenario where signal production does not depend on nutrient availability. This is our baseline scenario. Function $\gamma_2(C)$ describes the scenario

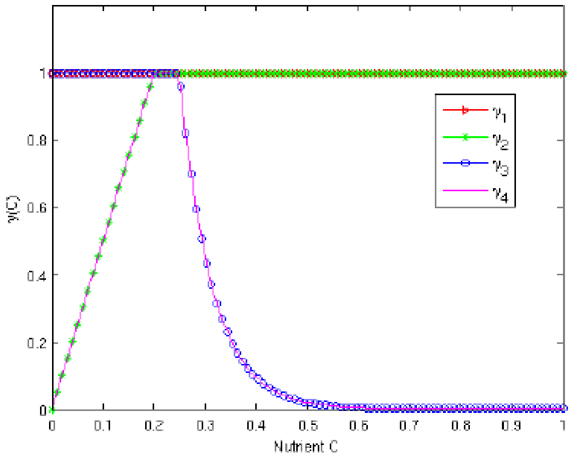

$$\gamma_1(C;k_2,k_3) = 1$$

$$\gamma_2(C;k_2,k_3) = \begin{cases} \frac{C}{k_2}, & \text{if } C \leq k_2 \\ 1, & \text{otherwise} \end{cases}$$

$$\gamma_3(C;k_2,k_3) = \begin{cases} 1, & \text{if } C \leq k_3 \\ -e^{-(C-k_3)log(\frac{eps}{2-k_3})}, & \text{otherwise} \end{cases}$$

$$\gamma_4(C;k_2,k_3) = \begin{cases} \frac{C}{k_2}, & \text{if } C \leq k_2 \\ 1, & k_2 < C \leq k_3 \\ -e^{-(C-k_3)log(\frac{eps}{2-k_3})}, & \text{otherwise} \end{cases}$$

Fig. 2: **The nutrient dependent functions $\gamma_i(C)$, $i = 1, 2, 3, 4$.** This function controls the production of quorum sensing molecule in the biofilm

where for low nutrient concentrations the signal production rate is proportional to the nutrient concentration whereas for larger nutrient concentrations the signal production rate is independent of the nutrient concentration, cf [2]. This reflects the argument that signal production is expensive and not affordable under unfavourable conditions. The opposite is the scenario described by $\gamma_3(C)$, where the signal production rate is highest during periods of low substrate availability, but it decreases if the substrate concentration increases. In this scenario signal production is a stress response. In $\gamma_4(C)$ these both effects are accounted for: the signal production rate is proportional to the substrate concentrations under conditions of low availability, it attains its maximum at intermediate substrate concentrations and declines as substrate becomes more and more plentiful, cf also [1].

For the simulation results shown in Fig.3, we fix $\tau = 120nM$ and $\eta_1 = 0.6d^{-1}$ while every other parameter used is listed on Table 1 in the main text. The choice of parameters in this simulation enables the investigation under limited substrate concentration for a biofilm with all the boundary conditions defined and described in the previous sections.

Note that the value of τ is chosen here higher than in our other simulations, in order to delay induction to allow the biofilm to grow bigger before dispersal starts and thus to enforce a lower nutrient concentration. A similar effect could have been achieved by reducing α and β.

On the other hand to enable the investigation under increased availability of nutrients we have considered also a microfloc, i.e. a biofilm without substratum. We prescribe a non-homogeneous Dirichlet boundary condition for the substrate on all the boundaries to ensure a constant supply of nutrients to the flocs across all domain boundaries i.e. $C = C_\infty$, where C_∞ is the bulk concentration value. Moreover, we pose a homogeneous Neumann boundary condition for the autoinducers on all the boundaries of the microfloc system to ensure that the signal molecules does not leave the system and is not diluted, i.e. $\partial_n A = 0$. For the biomass, we pose a homogeneous Dirichlet boundary condition on all the boundaries for the biomass i.e. $M = N = 0$, which allows the bacterial cells to leave from any of the boundaries. For the microfloc, we fix the signal threshold concentration $\tau = 50nM$ and the maximum dispersal rate $3.6d^{-1}$ while every other parameter used is as listed in Table 1 in the main text. Lumped output parameters for these simulations are presented in Figs.3 and 4.

We first focus on the biofilm (Fig.3). High nutrient concentrations are observed only initially and decline quickly as the biofilm grows. The concentration of the nutrient in the biofilm is not affected by the production of the quorum sensing signal molecule production rate which depends on the nutrient. Once the nutrient concentration drops below the concentration value k_2 we see a clear separation of curves for the total biomass density, biofilm size, fraction of dispersed cells and total amount of dispersed cells. The curves obtained for $\gamma_2(C)$ and $\gamma_4(C)$, which are sensitive to low concentration values are grouped together, as well as the curves for $\gamma_1(C))$ and $\gamma_3(C)$ which are not sensitive to low substrate concentrations. As long as the substrate concentration is above k_2 we do not detect a notable difference between the four curves.

In contrast to the biofilm, the autoinducer concentration level in the microfloc is sensitive to high nutrient concentration as shown in Fig.4. There is an increased autoinducer concentration level in the case of γ_3 and γ_4 when the nutrient concentration is above the concentration value k_2 and below k_3; these results are grouped together and are sensitive to high nutrient concentration. On the other hand, the autoinducer concentration is low for γ_1 and γ_2 which are grouped together and are insensitive to high nutrient concentration.

In general, we observe that the different concentration levels of the quorum sensing signal does not translate to cell dispersal.

References

[1] Bjorn MJ, Sokol PA, and Iglewski BH: **Influence of iron on yields of extracellular products in *Pseudomonas aeruginosa* cultures.** *J. Bacteriol* 1979, **138**:193-200.

[2] Bollinger N, Hassett DJ, Iglewski BH, Costerton JW, and McDermott TR: **Gene expression in *Pseudomonas aeruginosa*: evidence of iron override effects on quorum sensing and biofilm-specific gene regulation.** *J. Bacteriol.* 2001, **183**:1990-1996.

[3] D'Argenio DA, Calfee MW, Rainey, PB and Pesci EC. **Autolysis and autoaggregation in *Pseudomonas aeruginosa* colony morphology mutants**. *J. Bacteriol* 2002, **184**, 6481-6489.

[4] Hunt SM, Hamilton MA, Sears JT, Harkin G, and Reno J: **A computer investigation of chemically mediated detachment in bacterial biofilms.** *Microbiology* 2003, **149**:1155-1163.

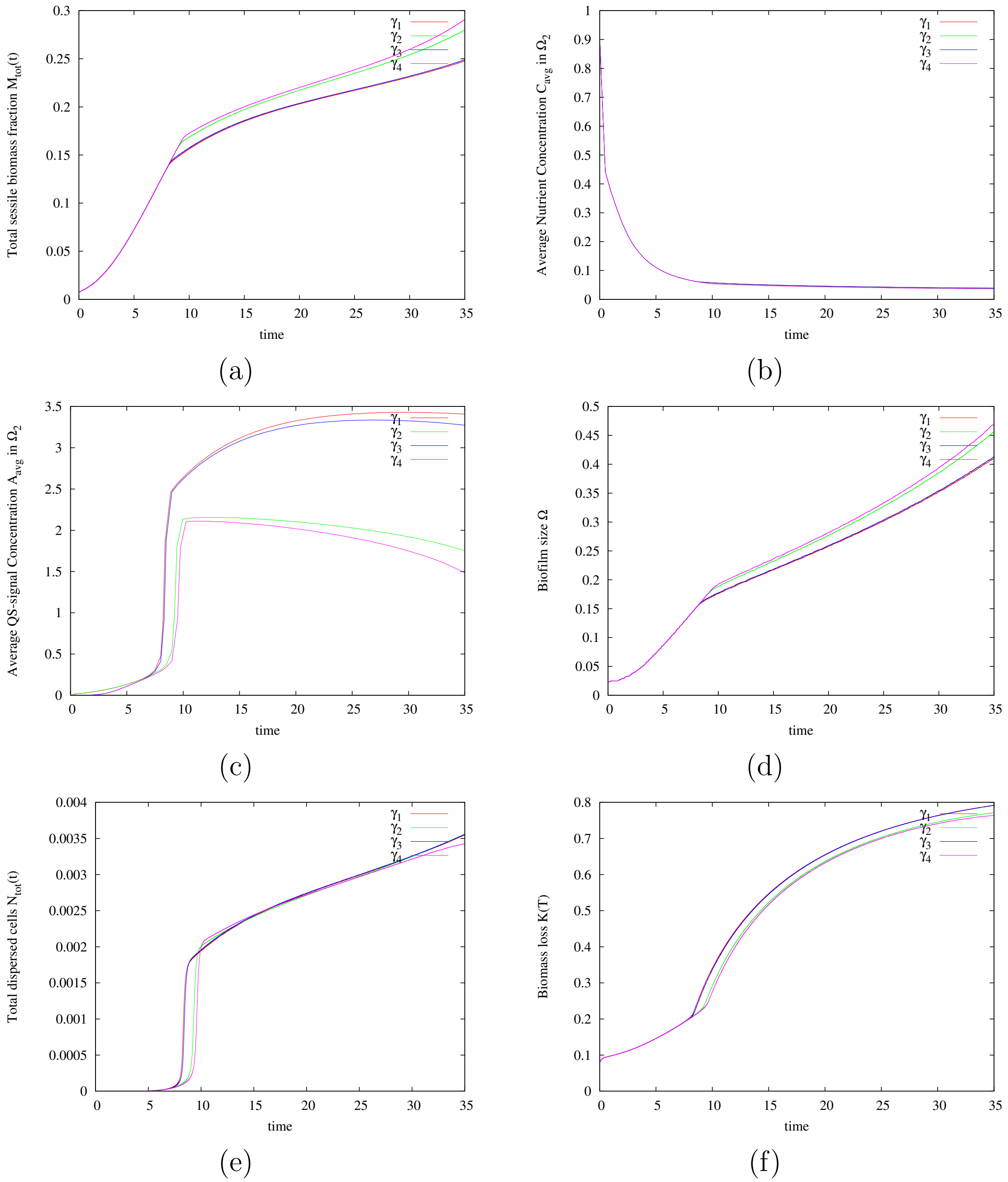

Fig. 3: **Temporal plots of simulations of a biofilm using different $\gamma_i(C)$.** Here we used $\tau = 120nM$, $\eta_1 = 0.6d^{-1}$. Shown are (a) total sessile biomass fraction M_{tot} in the biofilm , (b) average concentration of the nutrients C_{avg} in Ω_2, (c) average autoinducer concentration A_{avg} in Ω_2, (d) biofilm size ω, (e) total dispersed cells N_{tot}, (f) biomass loss $K(T)$ indicating the fraction of the produced biomass that are actually dispersed from the biofilm.

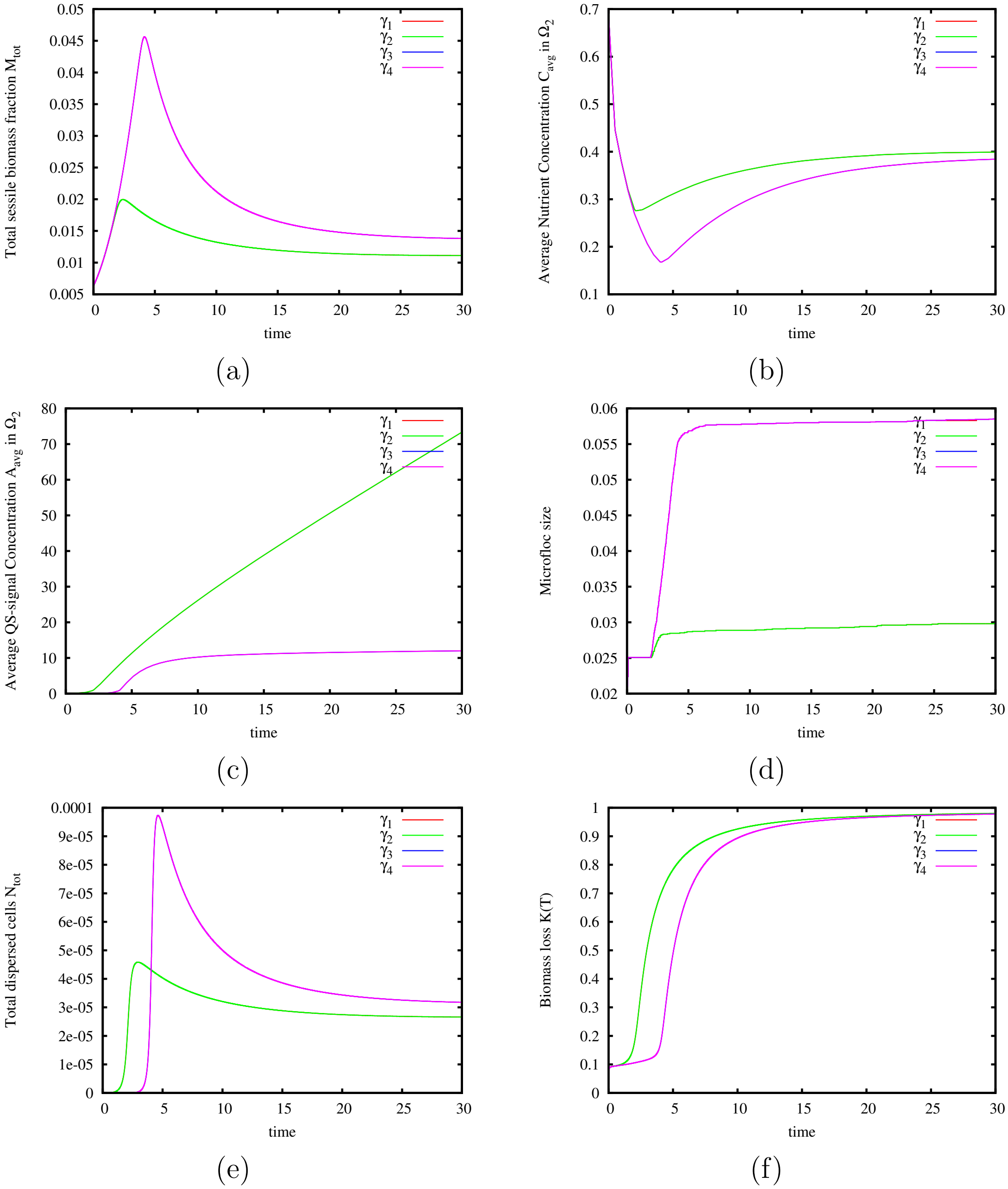

Fig. 4: **Temporal plots of simulations of a microfloc using different $\gamma_i(C)$.** Here we used $\tau = 50nM$, $\eta_1 = 3.6d^{-1}$. Shown are (a) total sessile biomass fraction M_{tot} in the microfloc, (b) average concentration of the nutrients C_{avg} in Ω_2, (c) average autoinducer concentration A_{avg} in Ω_2, (d) microfloc size Ω, (e) total dispersed cells N_{tot}, (f) biomass loss $K(T)$ indicating the fraction of the produced biomass that are actually dispersed from the microfloc. The curves of γ_1 and γ_2 (insensitive to high nutrient concentrations) are grouped together and appear as one curve; and so are those of γ_3 and γ_4 (sensitive to high nutrient concentrations).
